# Commentary: Primary Emotional Systems and Personality: An Evolutionary Perspective

**DOI:** 10.3389/fpsyg.2017.02143

**Published:** 2018-01-17

**Authors:** Simone Pollo

**Affiliations:** Department of Philosophy, Sapienza Università di Roma, Rome, Italy

**Keywords:** personality, character, evolution, ethics, sentimentalism

## Personality research and moral sentimentalism

In *Primary emotional systems and personality* Christian Montag and Jaak Panksepp analyze how emotional systems are involved into the development of basic personality into an evolutionary framework. They also stress the importance of such investigation for the promotion of human welfare in the context of psychiatric research and practice (Montag and Panksepp, 2017). Nonetheless, this field of study is of great relevance also for other disciplines and topics. Among these, philosophical ethics analyses can benefit from scientific explorations of human and non-human emotions and personality. Within the domain of philosophical ethics, in particular the perspective of moral sentimentalism can set a fruitful dialogue with those researches. From a methodological standpoint, moral sentimentalism (dating back to philosophers like David Hume and Adam Smith) is the ethical perspective more apt to fit into the framework of evolutionary biology (Kauppinen, 2017). As a matter of fact, Hume's philosophy anticipated some findings of Charles Darwin (and it is almost sure that the *Devil's Chaplain* has been a reader of *le bon David*: Huntley, 1972). Furthermore, methodology of sentimentalism is empirical and bottom-up like the the methods of Darwinian biology (Hume's aim in the *Treatise* was to build a “science of human nature”: Hume, 2000). From a more substantive point of view, sentiments, and character are central notions for moral sentimentalism and they are the very core of sentimentalist subjectivity and agency. For this reason in this brief commentary I will sketch three possible research lines that could be developed along the path traced by Montag and Panksepp in order to set a dialogue between their research and philosophical analysis of moral sentimentalism.

The first research topic can be generally described as the investigation of the development of emotions into sentiments and of personality into character. Sentiments and character are key concepts for moral sentimentalism, but they do not strictly overlap with emotions and personality as they are described and investigated by Montag and Panksepp (and in general by the research domain where their work is placed). Sentiments and character do not exactly overlap with emotions and personality because they are the product of social interactions, civilization processes, and reflective activities (Lecaldano, 2002). Emotions and personalities seem to be primigenial with respect to sentiments and character. If Montag and Panksepp are right in their claim that individual personalities are built upon primary emotions, then many questions should be faced by an empirically informed moral sentimentalism. Just one of them can be mentioned here: if personality traits are shaped by genes and early experiences what kind of processes can change and transform those traits? As a matter of fact, the topic of character development is crucial for moral sentimentalism and neuroscientific evidence sets a challenge for traditional views grounded on the idea that human beings have the power to somehow shape their own characters.

This first research topic is deeply intertwined with a second possible line of investigation. As already said, sentiments and character do not develop in isolation, but they are embedded in social and relational processes (Taylor, 2015). This idea is in tune with the evolutionary view endorsed by Montag and Panksepp, who stress the importance of relations for the development of primary emotions and then personality. Their remark about the importance of the study of epigenetic mechanisms highlights the potential role of environment for personality development. Then, research on personality should focus not just on the biological features of *Homo sapiens*, but at the same time on the environments where human beings are born and raised. From this perspective, a typical human environment presents particular features that are worth to be examined. One of this is morality. Within the whole of animal kingdom the life of the human animal is that most characterized by norms, rules, evaluations, approval practices, and so on. This feature is not a sign of human uniqueness as proved by observations of non-human social behavior (i.e., the brilliant ethological research of Frans de Waal, above all, De Waal, 1996). Nonetheless, it is beyond any doubt that the role played by morality in human environment is by far greater than for any other social species. Moral life can be regarded as one of the key traits of the niche constructed by *H. sapiens* in the course of evolution. According to the niche construction approach (Odling-Smee et al., 2003) features of the niche retroacts as a challenge for the selection of individuals living in it. With regard to morality as a key trait of the human niche and to personality/character research it could be fruitful to investigate how and in what degree social norms, rules, and values represent a selective pressure for emotions and personalities toward their development into sentiments and characters. A cooperation between neuroscience and philosophical ethics on this topic would represent a great advancement for the pursuit of the task of the naturalization of ethics (that is the full understanding of moral life in empirical terms without reference to any non-material cause). More specifically, research on morality as a niche and its effects on personality/character development could contribute also to understand the phenomenon of cultural variations of moral codes and norms.

Finally, a third research line could stem from this second one and could focus on moral education. According to sentimentalism moral education should mostly aim at the cultivation of given sentiments and at the development of a virtuous character (and this is why emotions and personality do not exactly overlap with sentiments and emotions). Highlighting the mechanism underlying personality development and its link to emotions can offer a solid ground for empirically tenable views on moral education. The interest of this last topic is not purely theoretical, but progress in this field could foster human welfare (as recommended by Montag and Panksepp themselves). Here it is not possible to argument, but it is very likely that an empirically informed sentimentalist view would strongly discourage rigoristic views of moral education and would confirm Hume's ideas about the so called “monkish virtues” (Davie, 1999). A scientifically grounded moral sentimentalism will confirm that sacrifice, self-denial, and mortification are vices rather than virtues as far as they interfere with human flourishing and promote neuroticism.

## Author contributions

The author confirms being the sole contributor of this work and approved it for publication.

### Conflict of interest statement

The author declares that the research was conducted in the absence of any commercial or financial relationships that could be construed as a potential conflict of interest.
